# Association between chronic diseases and falls among a sample of older people in Finland

**DOI:** 10.1186/s12877-020-01621-9

**Published:** 2020-06-26

**Authors:** Milla Immonen, Marianne Haapea, Heidi Similä, Heidi Enwald, Niina Keränen, Maarit Kangas, Timo Jämsä, Raija Korpelainen

**Affiliations:** 1 VTT Technical Research Centre of Finland Ltd. Kaitoväylä 1, P.O.Box 1100, FI-90571 Oulu, Finland; 2 Center for Life Course Health Research, University of Oulu, P.O. Box 5000, FI-90014 Oulu, Finland; 3 Department of Diagnostic Radiology, Oulu University Hospital, Kajaanintie 50, FI-90220 Oulu, Finland; 4 Medical Research Centre Oulu (MRC), Oulu University Hospital and University of Oulu, P.O. Box 5000, FI-90014 Oulu, Finland; 5 Information Studies, University of Oulu, P.O.Box 8000, FI-90014 Oulu, Finland; 6 Research Unit of Medical Imaging, Physics and Technology, University of Oulu, P.O. Box 5000, FI-90014 Oulu, Finland; 7 Department of Sports and Exercise Medicine, Oulu Deaconess Institute Foundation sr, Albertinkatu 16, FI-90100 Oulu, Finland

**Keywords:** Falls, Recurrent falls, Fall risk, Chronic diseases, Older adults, Geriatrics, Gerontology, Aging

## Abstract

**Background:**

Falls are a major problem for older people and recurrent fallers are especially prone to severe consequences due to falls. This study investigated the association between chronic conditions and falls.

**Methods:**

Responses from 872 older persons (age 65–98) to a health questionnaire were used in the analyses. Characteristics and disease prevalence between recurrent fallers, one-time fallers and non-fallers were compared. A hierarchical clustering method was applied to find combinations of chronic conditions that were associated with recent recurrent falling.

**Results:**

The results showed that recurrent fallers had a higher number of diseases (median 4, interquartile range, IQR = 2.0–5.0) compared to non-fallers (median 2, IQR = 1.0–3.0). Eight clusters were formed based on the data. The participants in the low chronic disease cluster were younger, more physically active, not frail, and had fewer geriatric conditions. Multiple chronic disease cluster participants were older, less physically active, overweight (body mass index, BMI > 30), at risk of malnutrition, and had more geriatric conditions. Significantly increased risk of recurrent falls relative to the low chronic cluster was found for respondents in the osteoporosis cluster and multiple chronic disease cluster (OR = 5.65, 95% confidence interval CI: 1.23–25.85, *p* = 0.026, and OR = 13.42, 95% CI: 2.47–72.96, *p* = 0.002, respectively). None of the clusters were associated with increased risk of one-time falling.

**Conclusions:**

The results implicate that the number of chronic diseases is related with risk of recurrent falling. Furthermore, the results implicate the potential of identifying certain combinations of chronic diseases that increase fall risk by analyzing health record data, although further studies are needed with a larger population sample.

## Background

Falls of older adults are globally a major health, economic and societal problem. One third of people over 65 years old fall at least once every year [1–5] and the number of falls per year increases with age and frailty level [6, 7]. Falls are associated with increased mortality, morbidity, reduced functioning, impaired quality of life and premature nursing home admissions [8]. Fear of falling may increase even after a non-injurious fall [9]. It can lead to a negative cycle where the activity of the person reduces, followed by a reduction in functionality. That, in turn, increases the need for help in daily activities [10, 11]. The world’s population is ageing rapidly; the number of people aged 65 or older is expected to grow from 524 million in 2010 to nearly 1.5 billion in 2050 [12]. Rapidly increasing ageing populations are a challenge to limited social and health care systems. At the same time, the costs resulting from falls and their consequences are increasing rapidly, and the importance of fall prevention grows. Screening for high fall risk persons consumes resources and public funding. Efficient methods for screening fall risk are needed to find the persons at most risk to target preventive actions towards them. Identifying recurrent fallers is especially important as they are more prone to severe consequences compared to non-recurrent fallers. For example, in a prospective study by Pluijm et al. 11.9% of recurrent fallers (at least two falls within 6 months) had a fall-related fracture compared to 3.4% of non-recurrent fallers [13]. One-time fallers (1 fall within 1 year) and recurrent fallers are reported to have distinct characteristics with respect to age, physiology, cognition, fear of falling and ability to perform daily activities [14–16].

Several chronic conditions have been identified to be associated with the higher prevalence of falling, including Parkinson’s disease [17, 18], heart failure [19], cognitive impairments [1, 20], diabetes [21], arthritis [22], depression [7, 23] and chronic kidney disease [24, 25]. In addition, conditions such as osteoporosis further increase the risk of fractures [26]. Along with the age, the prevalence of multiple chronic conditions increases [27–29], and earlier studies have identified co-occurrence of diseases and multi-morbidity patterns among older adults [30–32]. An association between history of falls and the number of diseases and the number of health conditions have been found, and the amount of falls rises with the number of diseases [32, 33]. Only a few studies have assessed, if there is a relationship between falls and multiple chronic conditions or multi-morbidity patterns [32, 34, 35]. Related to multi-morbidity studies, an agreement on which diseases should be included in the multi-morbidity assessment is missing. Based on a systematic literature review, Diederichs et al. recommended to include a minimum of 11 diseases (cancer, diabetes mellitus, depression, hypertension, myocardial infarction, chronic ischaemic heart disease, heart arrhythmias, heart insufficiency, stroke, chronic obstructive pulmonary disease, and arthritis) that are highly prevalent in the older population in the multi-morbidity assessment [36]. Moreover, Fortin et al. recommended that investigators designing future studies to assess the prevalence of multi-morbidity should consider the number of diagnoses to be assessed (with 12 or more frequent diagnoses of chronic diseases appearing ideal) and should attempt to report results for differing definitions of multi-morbidity (both 3 or more diseases and the classic 2 or more diseases) [37]. Recently, Griffith et al. concluded that the suitability of existing multi-morbidity measures for use in a specific research study depends on study-related factors such as purpose of the study, outcomes examined and preferences of the involved stakeholders [38]. In an extensive study using self-reported falls and disease data from 16,357 community dwelling older adults, Sibley et al. found falls to associate with a number of chronic conditions and with chronic disease patterns [32]. Another recent study with 149,876 respondents resulted in two “at risk” populations characterized by different chronic disease combinations and age. The authors concluded that these groups might benefit from different risk stratification and interventions [35]. In a sample of 2566 Swedish older adults, unhealthy lifestyle and high burden of chronic diseases were found to increase the risk of falling over long follow-up periods (5 and 10 years) [34]. Identifying diseases and possible individual clusters of certain conditions associated with risk of falling would help to develop solutions for screening those most at risk among older populations. Utilizing existing data saved on electronic health record provides a possibility for easy and cost-effective screening for high fall risk persons. Even though specific diseases have been identified to increase the risk of falling, and the number of chronic conditions have been linked to higher risk of falling, current fall prevention guidelines do not instruct measuring the amount of chronic conditions. The amount of earlier studies linking the amount or individual clusters of chronic conditions with higher fall risk is still very small [32, 34, 35]. Verifying the results with another sample help in improving fall prevention guidelines by recommending comprehensive fall assessment, or interventions aiming for fall prevention, among older adults with combinations of certain conditions that are connected with higher risk of falling. Thus, the aim of this study was to reveal the prevalence of falls and the association between number of chronic conditions and occurrence of recent falls, and the association between individual or multiple chronic conditions and occurrence of recent falls in a population-based sample of older people. In addition, demographic differences and lifestyle characteristics between non-fallers, one-time fallers and recurrent fallers were studied.

## Methods

A survey was conducted between November 2014 and January 2015 in Oulu, Finland. Oulu has a population of approximately 200,000 and it is Finland’s fifth largest city, located in Northern Finland. Of the population, 14% was aged 65 years or more at the end of 2014 [39]. The survey was part of a research study: Tailored Services for Elderly - Gamified remote service concept for promoting health of older people (GASEL-project, 2014–2016). Eleven volunteer seniors piloted the study questionnaire, and their feedback was used to make minor usability-related changes. A random sample of 1500 people living in Oulu was obtained from the Population Register Centre of Finland. The sampling criteria were: 1) born during 1.1.1900–31.12.1949 (aged 65–114 by the end of 2014); 2) spoke Finnish as a native language; and 3) had a permanent living address in Oulu in November 2014. A reminder and another copy of the questionnaire were sent to the non-responders 4 weeks after the first survey. The sample of 1500 persons represents approximately 5.5% of the over 65-year-old persons living in the city of Oulu and 918 persons (61.2%) responded to the survey (3.3% of the over 65-year-olds).

The study protocol was approved by the Ethics Committee of Human Sciences at the University of Oulu (statement 6/2014). The extensive questionnaire included items related to the participants’ socio-demographic background, perceived loneliness, stress, health, medication, social participation, personality, physical activity, functional ability, occurrence of recent falls, fear of falling, Internet and communications technology (ICT) use, and gaming. The questionnaire is available as supplementary material (Supplementary file 1, GASEL questionnaire (English version)).

To determine if the respondent had diseases diagnosed by a medical professional the question: “Do you currently have any of the following conditions as diagnosed by a medical professional?” and the answer options “yes” or “no” were used. The complete list of asked diseases can be found from the Supplementary file 1.

Frequency of recent falls was determined by the question: “Have you fallen during the last three (3) months? (A fall is defined as an event that results in a person coming to rest inadvertently on the ground or floor or other lower level).” The answer options were: “no” or “yes, ____ times.” Three month recall time was chosen, because in the target group the possibility of poorer cognitive function may lead to potential bias with longer recall time [40, 41].

The respondents were categorized according to their self-reported alcohol use into three categories: no alcohol, light drinker (1–3 drinks per week), moderate (4–7 drinks per week for women and 4–14 drinks per week for men), and heavy (≥8 drinks per week for women and ≥ 15 drinks per week for men) [42]. Smoking was categorized according to their selection of options: 1) I don’t smoke at all, 2) I quit smoking in year____, 3) I currently smoke ____ cigarettes a day, 4) I use snuff or other tobacco products other than cigarettes. Activity level was determined by the question: “How long are you active each day? (e.g. biking/walking, housework, walking to the store, exercise hobbies, etc.)? 1) < 1 hour per day, 2) 1-2 hours per day, and 3) > 2 hours per day” [43]. Sleep quality was determined by the question: “How do you feel about the quality of sleep you are currently getting (regardless of how much you sleep)?” and the answer options: 1) good 2) satisfactory, or 3) poor. “Can you rise from a chair independently without using your hands?” was used as a question to determine if the person was able to stand up from a chair. The answer options were: 1) I am able to rise independently without using my hands, 2) I am able to rise independently using my hands on first try, 3) I am able to rise using my hands after several tries, 4) I need minimal assistance to rise, and 5) I need moderate or maximal assistance to rise.” A modified SOF Frailty Index (Study of Osteoporotic Fractures) [44] described in our earlier study [45] was used to identify those with frailty symptoms. The modified items were: 1. Weight loss irrespective of intention to lose weight (if weight loss in 3 months is equal or more than 1 kg, score = 1), 2. “Can you rise from a chair independently without using your hands?” (if unable to rise from a chair, score = 1), and 3. Poor energy as identified by “Which of the following best describes how energetic you have felt in the last month?” (with the response “I feel moderately/very/extremely exhausted, tired or powerless”, score = 1). The subject’s status was categorized as frail if the SOF index was 2 or 3; intermediate if the SOF index was 1, and robust if the SOF index was 0. The Mini Nutritional Assessment (MNA) [46] includes seven questions about decrease in nutrition, weight loss, mobility, stress or acute illness, neuropsychological problems (dementia or depression), and Body Mass Index (BMI). Compared to the original MNA questionnaire, in our study weight loss was determined by the question: “In the last 3 months, has your weight 1) decreased by____ kg (if the decrease is more than 3 kg, score = 0, if the decrease is between 1 kg and 3 kg, score = 2), 2) stayed the same (score = 3), 3) increased by _____ kg (score = 3), and 4) I don’t know (score = 1).” Moreover, the question about psychological problems was modified: “Do you currently have any of the following conditions as diagnosed by a medical professional?” was used as a question to discover if the respondent had memory disorders (mild or moderate to severe), other neurological illness, or depression, with the answer options “yes” or “no.” In our modified MNA, 0 points is given if the respondent had moderate or severe memory disorder and/or depression, 1 point is given for mild memory disorder and 2 points is given if the respondent didn’t have memory disorders or depression. An individual is considered to have malnutrition if their total MNA score is 0–7 points, to be at risk of malnutrition at 8–11 points, and to have normal nutrition at 12–14 points.

### Statistical analysis

Analyses were performed using IBM SPSS Statistics for Windows, version 24 (IBM Corp., Armonk, NY, USA). The individual was classified as a non-faller, if he or she did not report any falls during the period of last 3 months. If the individual had fallen once during the last 3 months, he or she was categorized as a one-time faller and if the individual had fallen at least twice during the last 3 months he or she was categorized as recurrent faller. To describe the characteristics of the participants, frequencies with proportions and means with standard deviations were calculated separately for non-fallers, one-time-fallers and recurrent fallers. Chi-square test, analysis of variance (ANOVA) and Kruskal-Wallis test were used to analyze the statistical significance of the differences between non-fallers, one-time fallers and recurrent fallers in dichotomic, continuous, and non-normally distributed continuous variables, respectively. The background and lifestyle related variables were used to present the sample characteristics but they were not used in the cluster analysis.

If a respondent answered “yes” or “no” to at least one of the diseases but left the rest of the disease questions unanswered, the answers to the blank questions were interpreted as “no.” If the respondent didn’t choose any of the diseases, the respondent’s disease data were interpreted as missing. The number of chronic diseases was calculated. Chi-square test and Kruskal-Wallis test were used to compare the dichotomic disease variables and not normally distributed number of diseases -variable, respectively, between recurrent fallers, one-time fallers and non-fallers. Multinomial logistic regression was performed to analyze the relationship between the number of chronic diseases and falls.

To determine if there was an association between different combinations of chronic conditions and recent falling, a two-step statistical procedure was applied. To identify sub-groups of individuals according to their responses about currently having a chronic disease diagnosed by a doctor, a method of agglomerative hierarchical clustering with Ward’s linkage was used [47]. Following 15 diseases were included in clustering: diabetes, rheumatoid arthritis, coronary heart disease, elevated blood pressure, blood circulation problems in the brain, blood circulation problems in the legs, hypothyroidism, cancer, asthma, chronic obstructive pulmonary disease (COPD), heart failure, osteoporosis, mild, moderate or severe cognitive impairment, depression. Due to low prevalence, hyperthyroidism, Parkinson’s disease, liver diseases, eating disorder, kidney diseases, and mental illnesses (other than depression) were excluded in the cluster analysis, and due to vague definition and variety of diseases, the “other diseases” group was not included. Hierarchical clustering begins by treating each subject as an individual cluster. These clusters are gradually merged with the most similar other clusters until all subjects are included in a single cluster. This method produces a tree-like structure, a dendogram. The number of result clusters was chosen by visually inspecting the dendogram and agglomeration schedule coefficient and by analyzing the multiple morbidities inside the clusters. The cluster solution was validated by selecting a 50% random sample of the data and running the same agglomerative hierarchical clustering procedure in this sub-sample. The cluster memberships from the sub-sample were compared with the original memberships. Eventually, the relationship between chronic disease clusters and one-time and recurrent falls was studied by multinomial logistic regression, adjusted for age and gender. Similar methodology has been used in previous studies by Sibley et al. [32] and Ek et al. [34].

## Results

Eligible data on 872 participants were available. Of the respondents, 116 (13.3%) had fallen during the last 3 months. Seventy-one (8.4%) had fallen once, 27 (3.1%) two times, 10 (1.1%) three times, 3 (0.3%) four times and 5 (0.6%) more than five times. The characteristics of the study participants are presented in Table 1. Non-fallers were younger than the one-time or recurrent fallers (mean age 72.7, 76.6 and 76.0 years, respectively, *p* < 0.001). Categorization as frail was more common among recurrent fallers than non-fallers or one-time fallers (40.0, 5.7 and 7.6%, respectively, *p* < 0.001) and fallers were less frequently able to get up from a chair without using hands or without assistance than non-fallers or one-time fallers (39.7, 86.2, 79.4%, *p* < 0.001). Fallers had symptoms of malnutrition more often than non-fallers (37.5% for recurrent fallers, 22.5% for one-time fallers and 12.3% for non-fallers, *p* = 0.005). Recurrent fallers also had poor self-reported sleep quality more often than non-fallers or one-time fallers (19.5, 7.1 and 12.5%, respectively, *p* = 0.036).

**Table 1:** Study population characteristics

Variable		Non-faller (*n* = 756)	One-time faller (*n* = 71)	Recurrent faller (*n* = 45)	All (*n* = 872)	*p*-value
Age^a^		72.7 (6.5)	76.6 (6.9)	76.0 (8.2)	73.2 (6.7)	< 0.001
Gender	Male	319 (42.2%)	30 (42.3%)	20 (44.4%)	359 (43.4%)	0.957
	Female	437 (57.8%)	41 (57.7%)	25 (55.6%)	503 (57.7%)	
BMI		26.7 (4.7)	27.3 (4.0)	27.1 (6.0)	26.8 (4.7)	0.584
BMI	< 20	16 (2.2%)	2 (3.0%)	4 (9.1%)	22 (2.6%)	0.067
	20–24.9	267 (36%)	17 (25.4%)	13 (29.5%)	297 (34.8%)	
	25–29.9	323 (43.5%)	34 (50.7%)	17 (38.6%)	374 (43.8%)	
	> 30	136 (18.3%)	14 (20.9%)	10 (22.7%)	160 (18.8%)	
Alcohol use	No alcohol	337 (46.6%)	42 (63.6%)	21 (48.8%)	400 (48%)	0.153
	Light	255 (35.3%)	17 (25.8%)	12 (28%)	284 (34%)	
	Moderate	104 (14.4%)	6 (9.1%)	9 (21%)	119 (14%)	
	Heavy	27 (3.7%)	1 (1.5%)	1 (2%)	29 (3%)	
Physical activity, daily hours	< 1 h	113 (15.2%)	14 (19.7%)	12 (27.9%)	139 (16%)	0.076
	1–2 h	247 (33.2%)	25 (35.2%)	17 (39.5%)	289 (34%)	
	> 2 h	383 (51.5%)	32 (45.1%)	14 (32.6%)	429 (50%)	
Current smoker (> 1 cigarettes per day)		63 (8.5%)	1 (1.4%)	5 (12%)	69 (8%)	0.079
Sleep quality: Poor		52 (7.1%)	8 (12.5%)	8 (19.5%)	68 (8%)	0.036
Ability to rise from a chair without using hands		636 (86.2%)	54 (79.4%)	17 (39.5%)	707 (80%)	< 0.001
SOF-index (frailty)	Robust	509 (71.0%)	38 (57.6%)	14 (35%)	561 (68%)	< 0.001
	Intermediate	167 (23.3%)	23 (34.8%)	10 (25%)	200 (24%)	
	Frail	41 (5.7%)	5 (7.6%)	16 (40%)	62 (8%)	
MNA	Malnutrition	6 (1.3%)	0 (0.0%)	0 (0%)	6 (1%)	0.005
	Risk of malnutrition	58 (12.3%)	9 (22.5%)	9 (37.5%)	76 (14%)	
	Normal nutrition	406 (86.4%)	31 (77.5%)	15 (62.5%)	452 (85%)	

Values are numbers (percentage of number of participants having data on the variable in question) or means (standard deviation)

*BMI* Body mass index, *SOF* SOF Frailty Index (Study of Osteoporotic Fractures), *MNA* Mini Nutritional Assessment

^a^Median age (interquartile range, IQR): non-fallers 71 years (68–77), one-time fallers 78 (71–82), recurrent fallers 73 years (68–83)

The occurrence of chronic diseases and common geriatric conditions, urinary incontinence and dizziness, in the groups of fallers and non-fallers are shown in Table 2. The median number of diseases differed significantly in the groups of non-fallers, one-time fallers and recurrent fallers (medians 2, 3 and 4, respectively; *p* < 0.001). The frequency of rheumatoid arthritis, blood circulation problems in the brain and legs, cardiac failure, osteoporosis, Parkinson’s disease, moderate or severe cognitive impairment, mild cognitive impairment, depression, urinary incontinence, and dizziness were higher among recurrent fallers than non-fallers or one-time fallers (Table 2).

**Table 2:** Prevalence of diagnosed diseases among fallers and non-fallers

	All *n* = 872	Non-fallers *n* = 756	One-time fallers *n* = 71	Recurrent fallers *n* = 45	*P*-value
Number of diseases*	2.0 (2.0–3.0)	2 (1.0–3.0)	3 (1–3.3)	4 (2.0–5.0)	< 0.001*
At least one of the stated diseases	712 (83.6%)	611 (80.8%)	61 (85.9%)	40 (88.8%)	0.681
Diabetes	218 (25.6%)	180 (23.8%)	21 (23.9%)	17 (37.8%)	0.058
Rheumatoid arthritis	37 (4.3%)	27 (3.6%)	5 (7.0%)	5 (11.1%)	0.022
Coronary heart disease	161 (18.9%)	131 (17.3%)	17 (23.9%)	13 (28.9%)	0.062
Elevated blood pressure	417 (49.2%)	362 (47.9%)	32 (45.1%)	23 (51.1%)	0.72
Blood circulation problems in the brain	57 (6.7%)	43 (5.7%)	4 (5.6%)	10 (22.2%)	< 0.001
Blood circulation problems in the legs	138 (16.2%)	109 (14.4%)	14 (19.7%)	15 (33.3%)	0.002
hypothyroidism	109 (12.8%)	93 (12.3%)	10 (14.1%)	6 (13.3%)	0.898
Cancer	71 (8.4%)	58 (7.7%)	8 (11.3%)	5 (11.1%)	0.432
Asthma	102 (12.0%)	86 (11.4%)	8 (11.3%)	8 (17.8%)	0.388
COPD	32 (3.8%)	28 (3.7%)	3 (4.2%)	1 (2.2%)	0.861
Cardiac failure	81 (9.5%)	62 (8.2%)	8 (11.3%)	11 (24.4%)	0.001
Osteoporosis	62 (7.3%)	46 (6.1%)	8 (11.3%)	8 (17.8%)	0.004
Parkinson’s disease	11 (1.3%)	5 (0.7%)	2 (2.8%)	4 (8.9%)	< 0.001
Moderate or severe cognitive impairment	23 (2.7%)	14 (1.9%)	3 (4.2%)	6 (13.3%)	< 0.001
Mild cognitive impairment	106 (12.5%)	78 (10.3%)	16 (22.5%)	12 (26.7%)	< 0.001
Depression	41 (4.8%)	24 (3.2%)	7 (9.9%)	10 (26.7%)	< 0.001
Other disease	108 (12.7%)	90 (11.9%)	9 (12.7%)	9 (20.0%)	0.245
Liver disease	5 (0.6%)	5 (0.7%)	0 (0%)	0 (0%)	0.681
Kidney disease	15 (1.8%)	11 (1.5%)	1 (1.4%)	3 (7%)	0.028
Eating disorder	4 (0.5%)	3 (0.4%)	0 (0.0%)	1 (2.3%)	0.168
Other mental disorder	7 (0.8%)	6 (0.8%)	1 (1.4%)	0 (0.0%)	0.714
Urinary incontinence	215 (25.2%)	166 (22.0%)	25 (35.2%)	24 (55.8%)	< 0.001
Dizziness	226 (26.7%)	176 (24.0%)	25 (35.2%)	25 (56.8%)	< 0.001

Values are numbers (%), or medians (interquartile range, IQR), *COPD* chronic obstructive pulmonary disease

* Kruskal-Wallis. Mean number of diseases (standard deviation): non-fallers 2.17 (1.95), one-time fallers 2.74 (2.18), recurrent fallers 4.14 (2.52)

The number of chronic diseases and their relationship to recurrent falls are shown in Table 3. According to age and gender -adjusted multinomial logistic regression, the respondents with five or more chronic diseases significantly more often reported having two or more falls during the last 3 months than the participants with less chronic diseases. The odds ratio for having experienced recurrent falls was 11.70 (95% CI: 2.99–46.44) with five chronic diseases and 8.60 (95% CI: 2.10–35.17) with six or more chronic diseases compared to no chronic diseases.

**Table 3:** Association between number of chronic diseases and falls according to multivariable logistic regression analysis adjusted for age

	Variable	OR (95% CI)	*P-value*
One-time faller	No chronic diseases	reference	
	One chronic disease	0.392	0.392
	Two chronic diseases	0.526	0.526
	Three chronic diseases	0.424	0.424
	Four chronic diseases	0.770	0.770
	Five chronic diseases	0.616	0.616
	Six or more chronic diseases	0.529	0.529
	Age 65–74	reference	
	Age 75–84	4.48 (2.56–7.83)	< 0.001
	Age ≥ 85	2.78 (1.03–7.48)	0.044
	Gender	0.98 (0.59–1.65)	0.951
Recurrent faller	No chronic diseases	reference	
	One chronic disease	0.72 (0.14–3.64)	0.692
	Two chronic diseases	1.30 (0.30–5.60)	0.723
	Three chronic diseases	2.92 (0.76–11.19)	0.116
	Four chronic diseases	1.82 (0.35–9.47)	0.474
	Five chronic diseases	11.7 (2.99–46.44)	0.000
	Six or more chronic diseases	8.60 (2.10–35.17)	0.003
	Age 65–74	reference	
	Age 75–84	1.00 (0.47–2.13)	0.947
	Age ≥ 85	2.07 (0.82–1.07)	0.122
	Gender (female)	1.05 (0.54–1.05)	0.870

*OR* Odds ratio, *CI* Confidence Interval

Agglomerative hierarchical clustering with Ward’s linkage resulted in eight clusters. The formed clusters are shown in Table 4. Cluster 1 participants had a low number of chronic conditions. Cluster 2 participants had the highest prevalence of elevated blood pressure. Cluster 3 participants had coronary artery disease, and heart failure as well as diabetes. Cluster 4 participants had asthma and COPD, and Cluster 5 participants hypothyroidism and diabetes. Cluster 6 in the main included participants with osteoporosis, but the prevalence of elevated blood pressure was not seemingly high compared to the other clusters. Cluster 7 consisted of participants with blood circulation problems in the legs. The multiple chronic disease Cluster 8 included 32 participants who had a higher prevalence of multiple chronic conditions; elevated blood pressure, diabetes, coronary heart disease, heart failure, cognitive impairments, blood circulation problems in brain and legs, asthma, COPD and rheumatoid arthritis. The formed clusters were labeled after the disease, which prevalence in the cluster exceeds the prevalence in the total sample and the prevalence in the other clusters, except for the elevated blood pressure. In the split sample for validation, agglomerative hierarchical clustering formed similarly eight clusters, of which one cluster included healthy participants and one cluster included participants with multiple chronic conditions.

**Table 4:** Chronic diseases by cluster

Cluster	1	2	3	4	5	6	7	8	Total
Label	Low chronic disease	Elevated blood pressure	Coronary artery disease	Asthma	Hypothyroidism	Osteoporosis	Blood circulation problems in legs	Multiple chronic disease	
N	138	227	100	64	59	176	66	32	862
*Diseases*									
Diabetes	0.7%	**33.5%**	**42.0%**	12.5%	**39.0%**	17.6%	22.7%	**84.4%**	25.9%
Rheumatoid arthritis	0.0%	**7.5%**	2.0%	**9.4%**	0.0%	4.0%	0.0%	**12.5%**	4.2%
Coronary artery disease	0.0%	5.3%	**88.0%**	17.2%	3.4%	9.1%	**22.7%**	**59.4%**	18.9%
Elevated blood pressure	1.4%	**69.6%**	**58.0%**	**57.8%**	49.2%	48.3%	43.9%	**87.5%**	49.4%
Blood circulation problems in the brain	0.0%	**8.8%**	6.0%	6.3%	0.0%	7.4%	1.5%	**43.8%**	6.7%
Blood circulation problems in the legs	0.0%	3.5%	5.0%	15.6%	10.2%	11.4%	**100.0%**	**78.1%**	16.2%
hypothyroidism	0.0%	4.0%	**15.0%**	**14.1%**	**98.3%**	5.7%	7.6%	**28.1%**	13.3%
Cancer	0.0%	**18.1%**	2.0%	1.6%	5.1%	5.7%	7.6%	**31.3%**	8.4%
Asthma	0.0%	1.8%	6.0%	**95.3%**	6.8%	9.1%	4.5%	**40.6%**	12.4%
COPD	0.0%	1.3%	1.0%	**17.2%**	1.7%	2.8%	1.5%	**31.3%**	3.7%
Heart failure	0.0%	4.8%	**37.0%**	4.7%	5.1%	2.8%	6.1%	**71.9%**	10.0%
Osteoporosis	0.0%	0.9%	0.0%	1.6%	**8.5%**	**27.8%**	3.0%	**18.8%**	7.5%
Moderate or severe cognitive impairment	2.9%	0.9%	2.0%	1.6%	1.7%	**5.7%**	0.0%	**12.5%**	2.8%
Mild cognitive impairment	0.0%	**21.6%**	11.0%	9.4%	8.5%	8.5%	9.1%	**53.1%**	12.6%
Depression	0.0%	2.6%	**6.0%**	**9.4%**	**5.1%**	**8.0%**	0.0%	**21.9%**	4.9%

Values are percentages among the cluster. Highlighted values represent conditions with higher prevalence in the cluster than in the total sample. *COPD* chronic obstructive pulmonary disease

The identified clusters also differed with regard to demographic characteristics and geriatric conditions, as shown in Table 5. The participants in Cluster 1 were younger, more physically active, more frequently non-frail, and had smaller prevalence of geriatric conditions such as urinary incontinence and dizziness. In contrast, multi-morbid Cluster 8 participants were older, less physically active, more often overweight (BMI > 30), at risk of malnutrition, and had higher prevalence of geriatric conditions such incontincence and dizziness.

**Table 5:** Study group characteristics by cluster

Cluster	1	2	3	4	5	6	7	8	Total
Label	Low chronic disease	Elevated blood pressure	Coronary artery disease	Asthma	Hypothyroidism	Osteoporosis	Blood circulation problems in legs	Multiple chronic disease	
*Age*									
65–74	**81.9%**	60.8%	45.0%	**78.1%**	**67.8%**	59.7%	60.6%	21.9%	62.4%
75–84	15.2%	**33.9%**	**44.0%**	17.2%	27.1%	**30.7%**	**33.3%**	**53.1%**	30.4%
≥ 85	2.9%	5.3%	**11.0%**	4.7%	5.1%	**9.7%**	6.1%	**25.0%**	7.2%
*BMI*									
< 20	**2.9%**	0.9%	**3.1%**	1.6%	1.8%	**4.6%**	1.6%	0.0%	2.4%
20–24,9	**38.0%**	30.1%	29.6%	**44.4%**	26.3%	**38.2%**	**38.1%**	28.1%	34.3%
25–29,9	**50.4%**	**48.4%**	**50.0%**	31.7%	**54.4%**	35.3%	39.7%	31.3%	44.1%
> 30	8.8%	**20.5%**	17.3%	**22.2%**	17.5%	**22.0%**	20.6%	**40.6%**	19.2%
*Daily activity*									
< 1 h	13.1%	15.7%	**20.6%**	**20.6%**	**17.2%**	**18.6%**	6.3%	**32.3%**	16.8%
1–2 h	25.5%	**38.6%**	29.9%	31.7%	**43.1%**	33.1%	**35.9%**	32.3%	33.7%
> 2 h	**61.3%**	45.7%	49.5%	47.6%	39.7%	48.3%	**57.8%**	35.5%	49.5%
Urinary incontinence	0.7%	8.4%	**25.0%**	15.6%	**33.9%**	**61.9%**	10.6%	**75.0%**	24.9%
Dizziness	0.0%	15.0%	26.0%	17.2%	8.5%	**62.5%**	**30.3%**	**71.9%**	26.6%
*SOF-index*									
Robust	**84.2%**	65.4%	60.4%	**75.0%**	**72.7%**	62.2%	**72.7%**	33.3%	67.9%
Intermediate	13.5%	**25.1%**	**30.8%**	21.9%	20.0%	**28.7%**	21.2%	**30.0%**	23.8%
Frail	2.3%	**9.5%**	**8.8%**	3.1%	7.3%	**9.1%**	6.1%	**36.7%**	8.2%
*MNA Index*									
Malnutrition	0.9%	**1.6%**	0.0%	**2.4%**	0.0%	1.0%	**2.4%**	0.0%	1.1%
Risk of maln.	7.0%	12.5%	6.1%	**19.0%**	9.4%	**23.0%**	**14.3%**	**42.1%**	14.3%
Normal nutr.	**92.1%**	**85.9%**	**93.9%**	78.6%	**90.6%**	76.0%	83.3%	57.9%	84.6%

Percentages among the clusters. *BMI* Body Mass Index, *SOF* SOF Frailty Index (Study of Osteoporotic Fractures), *MNA* Mini Nutritional Assessment. Highlighted values represent conditions with higher prevalence in the cluster than in the total sample

Multinomial logistic regression analyses (Table 6) showed that Cluster 6 (osteoporosis, OR = 5.65, 95% CI: 1.23–25.85, *p* = 0.026) and Cluster 8 (multiple chronic disease, OR = 13.42, 95% CI: 2.47–72.96, p = 0.002) were significantly associated with recurrent falling (≥2 falls during the last 3 months) relative to Cluster 1 (low-chronic). None of the clusters was associated with higher risk of one-time falling. In the validation sample, cluster 8 (multiple chronic disease), was also significantly associated with recurrent falling (*p* = 0.006) and none of the clusters formed with the validation sample were associated with one-time falling.

**Table 6:** Association between cluster of chronic conditions and one-time falls and recurrent falls according to multinomial logistic regression analyses adjusted for age and sex. Results presented as odds ratios (OR) and 95% confidence intervals (95% CI)

	Variable	OR (95% CI)	*p-value*
1 fall	1 Low-chronic	reference	
	2 Elevated blood pressure	0.87 (0.35–2.16)	0.766
	3 Coronary heart disease	1.41 (0.53–3.76)	0.492
	4 Asthma	0.73 (0.18–2.91)	0.650
	5 Hypothyroidism	1.87 (0.62–5.66)	0.268
	6 Osteoporosis	1.36 (0.55–3.37)	0.512
	7 Blood circulation problems in the legs	0.75 (0.21–2.69)	0.662
	8 Multi-chronic	0.60 (0.11–3.11)	0.539
	Age 65–74	reference	
	Age 75–84	4.96 (2.81–8.74)	**< 0.001**
	Age ≥ 85	3.12 (1.18–8.29)	**0.022**
	Gender (male)	1.05 (0.62–1.78)	0.871
2 or more falls	1 Low-chronic	reference	
	2 Elevated blood pressure	1.80 (0.35–9.13)	0.480
	3 Coronary heart disease	4.93 (0.98–24.90)	0.054
	4 Asthma	1.03 (0.09–11.60)	0.984
	5 Hypothyroidism	2.60 (0.35–19.22)	0.350
	6 Osteoporosis	5.65 (1.23–25.85)	**0.026**
	7 Blood circulation problems in the legs	3.12 (0.50–19.37)	0.223
	8 Multi-chronic	13.42 (2.47–72.96)	**0.003**
	Age 65–74	reference	
	Age 75–84	1.186 (0.559–2.515)	0.657
	Age ≥ 85	3.076 (1.245–7.600)	**0.015**
	Gender (male)	1.072 (0.547–2.101)	0.839

In the multinomial regression, the reference group is non-fallers and the 2 comparison groups are persons with 1 fall and persons with 2 or more falls. Low-chronic group, age group 65–74 and female gender are used as reference groups

## Discussion

This study examined the association between chronic diseases and falls in a sample of older people (age 65–98) and demonstrated that fall incidence among community-dwelling older people is highly linked to presence of chronic disease. Individuals with five, six or more chronic diseases had significantly higher fall rates compared to those without diseases and several individual chronic diseases were associated with increased fall rates. The study showed, by clustering chronic diseases, that combinations of chronic diseases play a role in falls. The group with multiple chronic conditions had significantly higher risk of recurrent falling relative to the group of low chronic disease subjects. Osteoporosis did not cluster clearly with other diseases, but the group with osteoporosis was associated with increased risk of recurrent falls when compared with the group with low chronic disease.

The prevalence of recurrent fallers increased with age, which aligns with previous findings in the literature [3, 4, 6, 8, 48]. We also found that the percentage of subjects who reported poor sleep quality is higher in the recurrent fallers group and one-time fallers group compared to non-fallers (19.5, 12.5, 7.1%, respectively), and similar findings were made regarding frailty according to the modified SOF-index (40, 7.6 and 5.7%), and malnutrition according to MNA (37.5, 22.5 and 12.3%). The prevalence of recurrent fallers is higher among older adults with self-reported urinary incontinence and dizziness, which supports the findings in other studies [30, 49]. The number of falls differed between subjects with diabetes, rheumatoid arthritis, blood circulation problems in the brain and legs, cardiac failure, Parkinson’s disease, osteoporosis, moderate or severe cognitive impairment, mild cognitive impairment, depression, dizziness, and urinary incontinence compared to subjects without the specific condition. These diseases and geriatric conditions have been found to increase fall risk also in previous studies [1, 7, 17–26].

Our results showed a correlation between multiple chronic diseases and recurrent falls. Compared to the no disease group with age adjustment, increase in the risk of recurrent falls was found in the group having five or more diseases. This finding is supported by the findings in previous studies [32, 50]. We also found by clustering diseases that the multiple chronic disease group had a significantly higher risk of having fallen recurrently during the last 3 months than the group of low chronic diseases. Subjects in the osteoporotic cluster also had a high prevalence of elevated blood pressure and were more likely to have fallen during the last 3 months. It is also important to note that not all clusters were associated with high fall risk and none of the disease clusters was significantly associated with higher risk of one-time falling. This finding supports the previous findings of different characteristics of one-time fallers and recurrent fallers [14–16]. All clusters, except the low chronic cluster, had a notable percentage of subjects with elevated blood pressure (43.9–87.5%). The highest prevalence was in the multiple chronic disease cluster and elevated blood pressure was, in fact, the most common disease within that cluster. Our results partly align with previous studies. Sibley et al. found in their Canadian population-based study that clusters of hypertension and COPD had higher risk of falling than the low chronic diseases cluster [32]. In an earlier study by Paliwal et al., a positive association was found between the number of falls and certain diseases; heart attack, angina, stroke, asthma, COPD, chronic kidney disease, arthritis, depression, and diabetes [35]. The combinations of diseases associated with higher prevalence of falling differ between our study and the studies by Sibley et al. and Paliwal et al. This may be due to slightly different questionnaires used to collect information on medical history and diagnoses and different lists of asked diseases. In addition, we had a relatively low sample size compared to previous two studies and we had to exclude some of the diseases from clustering due to low prevalence of these diseases in the total sample. Similarly as in the previous studies, we did not collect any information on the treatment of the diseases, which may have had an impact on the results. National differences may also exist. In another study, Ek et al. used sociodemographic and lifestyle-related characteristics in forming clusters and had a follow-up period of 5 years. Their finding was that participants with unhealthy lifestyle and a high burden of chronic disease had higher risk of falling over a longer period of follow-up (5 and 10 years) compared to those in the reference group [34].

The strength of this study is its population-based sample and analysis as well as its rather unique approach of clustering diseases and studying their association with falls. However, some limitations of this study need to be taken into account. Firstly, the questionnaire was completed only once and no follow-up period was included. Furthermore, we have no information if the diagnosis of the certain chronic disease was given before or after the occurrence of falls, and no specific information about the diseases, such as the type or location of cancers. We also did not study how severe the diseases were or how the diseases or conditions were controlled or medicated. Cognitive impairments may also affect the results. The prevalence of certain diagnosis of some pathologies such as dementia or osteoporosis may be underestimated. Some diseases have low prevalence and, for example, Parkinson’s disease was not included in the cluster analysis even though it is commonly known to produce a high risk of falls. The three-month recall time may also lead to absence of actual recurrent falls in our study sample and the actual amount of recurrent fallers may be higher [51]. A larger population sample, for example from health records, would give more information about the chronic diseases and falls and show the potential of using health records in automatic screening of persons with high fall risk.

Our study gives promising results about the association between diseases and falls and further implicates that using data sources such as patient health records for screening for high fall risk individuals is important. In addition, the use of machine learning methods in health records for screening for high-risk persons is still unexploited. Future studies should utilize available data sources from patient records to study the association between combination of diagnoses and falls. Studies with large data sets should consider utilizing machine learning when analyzing the relationship between clusters of chronic conditions and falls.

## Conclusions

This study supports the earlier findings that risk of recurrent falls increases with age. The results further indicate that persons with certain combinations of chronic diseases may have elevated fall risk and the risk is highest with multiple chronic conditions. Multiple chronic disease cluster participants were, in general, older, less physically active, overweight, at risk of malnutrition, and had more geriatric conditions compared to the low chronic cluster. In addition to the multiple chronic disease cluster, clusters where osteoporosis and elevated blood pressure were the defining factors had significantly higher risk of falls. Notably, persons with five or more chronic conditions reported significantly more often having fallen at least two times within the last 3 months. Although further studies with a larger population are needed, these results implicate the potential of identifying high-risk persons by analyzing health record data. Finally, the results show that certain combinations of chronic diseases, especially multiple chronic conditions, increase the risk of recurrent falls.

## Supplementary information

**Additional file 1.** GASEL questionnaire (English version). Translated version of the study questionnaire. Original language of the questionnaire is Finnish. Linguistic validation between Finnish and English versions has not been performed.

## Data Availability

Data cannot be shared publicly because it includes sensitive personal health related data. Data are available from the University of Oulu Institutional Data Access (NFBC Project center: projectcenter@oulu.fi) for researchers who meet the criteria for access to confidential data. The possibilities for data sharing (anonymized or using pseudonyms) with researchers outside project consortium will be evaluated case by case by the NFBC Scientific Committee.

## Abbreviations

BMI: Body Mass Index
CI: Confidence Interval
COPD: Chronic Obstructive Pulmonary Disease
IQR: Interquartile range
MNA: Mini Nutritional Assessment
OR: Odds ratio
SOF: Study of Osteoporotic Fractures
