# Supplementary material for: Association between chronic diseases and falls among a sample of older people in Finland
Source: BMC Geriatr. 2020 Jun 26;20:225. doi: 10.1186/s12877-020-01621-9 (PMC7318483; doi:10.1186/s12877-020-01621-9)
Supplement: Supplementary file 1 — Additional file 1. GASEL questionnaire (English version). Translated version of the study questionnaire. Original language of the questionnaire is Finnish. Linguistic validation between Finnish and English versions has not been performed. [file 12877_2020_1621_MOESM1_ESM.docx]

G

Please circle the number of each question or each table line that best describes your situation or opinion unless otherwise instructed.

# background information

## what year were you born?

Year ______

## gender

1 Man

2 Woman

## height and weight

Height ______ cm

Weight _____ kg

## In the last 3 months, has your weight

1 Decreased by____ kg

2 Stayed the same

3 Increased by _____ kg

4 I don't know

# Education, occupation and work history

## What is your level of education?

1 Less than primary education

2 Primary education

3 High school or college

4 University degree or similar

## What type of work have you done in the past??

|  |  | How would you best describe the physical activity level at this occupation? | | |
| --- | --- | --- | --- | --- |
| Occupation | Years in this occupation | Sedentary or light activity | Moderate activity | Heavy activity |
|  |  | 1 | 2 | 3 |
|  |  | 1 | 2 | 3 |
|  |  | 1 | 2 | 3 |
|  |  | 1 | 2 | 3 |
|  |  | 1 | 2 | 3 |

## At what age did you retire or otherwise leave the workforce?

1 _____ years of age

2 I have not yet left the workforce

# Habitation and marital status

## Which of the following best describes your current form of residence?

1 Single-family house, detached, semi-detached or row house

2 Multi-story apartment building with elevator

3 Multi-story apartment building without elevator

4 Assisted living building, rehabilitation center, or nursing home

## What is your current marital status?

1 Married, in a registered partnership or cohabiting
2 Single
3 Widowed
4 Divorced

## Does your household include persons other than yourself and your potential spouse?

1 No

2 Yes, who? ___________________________________________________________________________

## next questions are about family caregiving

|  | Yes, official contract | Yes, no official contract | Not anymore, but I have been before | No |
| --- | --- | --- | --- | --- |
| Are you a family caregiver? | 1 | 2 | 3 | 4 |
| Do you have a family caregiver? | 1 | 2 | 3 | 4 |

|  | Daily or almost daily | Weekly | Less frequently | Not at all |
| --- | --- | --- | --- | --- |
| Do you regularly care for a disabled family member or other loved one outside of your home? | 1 | 2 | 3 | 4 |
| Do family members or other loved ones who live elsewhere regularly come to care for you? | 1 | 2 | 3 | 4 |

## In the last month (30 days), how much burden have you experienced from being a caregiver of a family member or loved one?

Circle the number that best describes the burden. 0 indicates that you experience no burden, and 10 means extreme burden.

| 0 | 1 | 2 | 3 | 4 | 5 | 6 | 7 | 8 | 9 | 10 |
| --- | --- | --- | --- | --- | --- | --- | --- | --- | --- | --- |

## Is your household income sufficient for your needs?

1 Yes, very well

2 Yes, reasonably well

3 Barely

4 No (I am using up my savings or being supported by my friends and family)

# Fitness and physical functioning

## Which of the following best describes your current mobility?

1 Bed or chair bound

2 Able to get out of bed/chair, but do not go out

3 Go out

## Do you use mobility aids? Select all that apply.

|  | Do not use | Outdoors | Indoors |
| --- | --- | --- | --- |
| Cane | 1 | 2 | 3 |
| Walker or rollator | 1 | 2 | 3 |
| Wheelchair | 1 | 2 | 3 |
| Other, what: | 1 | 2 | 3 |

## Can you rise from a chair independently and without using hands?

1 I am able to rise independently and without using hands

2 I am able to rise independently using hands, on first try

3 I am able to rise using hands after several tries

4 I need minimal aid to rise

5 I need moderate or maximal assist to rise

## Which of the following best describes how energetic you have felt in the last month?

1 I feel healthy and vital

2 I feel slightly exhausted, tired or powerless

3 I feel moderately exhausted, tired or powerless

4 I feel very exhausted, tired or powerless

5 I feel extremely exhausted, tired or powerless

## How long are you active each day? (eg. biking/walking, housework, walking to the store, exercise hobbies, etc?)?

1 Under 1 hour per day

2 1-2 hours per day

3 Over 2 hours per day

## How often do you participate in leisure time exercise?

|  | Once a month or less | 2-3 times per month | Once a week | 2-3 times a week | 4-6 times a week | Daily |
| --- | --- | --- | --- | --- | --- | --- |
| Light activity (you don't become short of breath or sweating) | 1 | 2 | 3 | 4 | 5 | 6 |
| Moderate or heavy activity (you become short of breath and sweat at least some) | 1 | 2 | 3 | 4 | 5 | 6 |

## For how long do you exercise at one time??

|  | Not at all | Under 20 minutes | 20-39 minutes | 40-59 minutes | 1-1,5 hours | Over 1,5 hours |
| --- | --- | --- | --- | --- | --- | --- |
| Light activity (you don't become short of breath or sweating) | 1 | 2 | 3 | 4 | 5 | 6 |
| Moderate or heavy activity (you become short of breath and sweat at least some) | 1 | 2 | 3 | 4 | 5 | 6 |

## How many hours do you sit on a weekday, on average? (Write 0, if not at all)

At home, for example watching TV or at the computer _____ hours _____ minutes

In a vehicle _____ hours _____ minutes

Elsewhere_______________________________ _____ hours _____ minutes

# Health history

## How do you rate your current health?

1 Very good

2 Good

3 Not good or bad

4 Bad

5 Very bad

## Do you feel that your physical performance is good for your age?

1 My physical performance is much better than most people my age.

2 My physical performance is slightly better than most people my age.

3 My physical performance is like that of most people my age.

4 My physical performance is slightly weaker than most people my age.

5 My physical performance is much weaker than most people my age.

## How much do you currently smoke?

1 I don't smoke at all

2 I quit smoking in year____

3 I currently smoke ____ cigarettes a day

4 I use snuff or other tobacco products other than cigarettes?

## Do you currently use any alcoholic beverages even occasionally? (eg. beer, cider, mild wines, wines, or spirits).

1 I do not

2 Yes, I do, on average _____ units per week. One unit of alcohol is equivalent to a standard glass of beer (285ml), a single measure of spirits (30ml), a medium-sized glass of wine (120ml), or 1 measure of an aperitif (60ml).

## How often do you brush your teeth?

1 Never or hardly ever

2 Occasionally throughout the week

3 Once a day

4 Twice a day or more

# Diseases and conditions

## Do you currently have any of the following conditions as diagnosed by a medical professional?

|  | Yes | No |
| --- | --- | --- |
| A liver disease | 1 | 2 |
| A kidney disease | 1 | 2 |
| Diabetes: | 1 | 2 |
| Diet treatment | 1 | 2 |
| Tablet treatment | 1 | 2 |
| Insulin treatment | 1 | 2 |
| Rheumatoid arthritis | 1 | 2 |
| Coeliac disease | 1 | 2 |
| Coronary heart disease | 1 | 2 |
| Elevated blood pressure | 1 | 2 |
| Blood circulation problems in the brain (eg. stroke) | 1 | 2 |
| Blood circulation problems in the legs (eg. varicose vein) | 1 | 2 |
| Hypothyreosis | 1 | 2 |
| Hyperthryreosis | 1 | 2 |
| Cancer | 1 | 2 |
| Lactose intolerance or milk allergy | 1 | 2 |
| Asthma | 1 | 2 |
| Chronic obstructive lung disease (COPD) | 1 | 2 |
| Heart failure | 1 | 2 |
| Osteoporosis | 1 | 2 |
| Parkinson's disease | 1 | 2 |
| Moderate to severe memory disorder | 1 | 2 |
| Mild memory disorder | 1 | 2 |
| Other neurological illness, what: | 1 | 2 |
| Eating disorder (eating behavior) | 1 | 2 |
| Depression | 1 | 2 |
| Other mental disorder, what: | 1 | 2 |
| Other, what: | 1 | 2 |

## Do you experience the following symptoms?

|  | Yes | No |
| --- | --- | --- |
| Urinary incontinence | 1 | 2 |
| Dizziness | 1 | 2 |

# medications

## How many different medications do you regularly use per day?

______ different medications

## Do you regularly use any of the following medication?

|  | Yes | No |
| --- | --- | --- |
| Blood thinning medication | 1 | 2 |
| Blood pressure medication | 1 | 2 |
| Cardiac nitroglycerin | 1 | 2 |
| Cholesterol medication | 1 | 2 |
| Osteoporosis medication | 1 | 2 |
| Hormone replacement therapy | 1 | 2 |
| Oral cortisone treatment | 1 | 2 |
| Sleep medication | 1 | 2 |
| Depression medication | 1 | 2 |
| Psychosis medication | 1 | 2 |
| Memory medication | 1 | 2 |
| Strong pain medication (e.g. opiates; **not** over-the-counter medications) | 1 | 2 |

# Fracture and fall history

|  | Yes | No |
| --- | --- | --- |
| Have you ever had a hip fracture?? | 1 | 2 |
| Have you ever had a bone fracture in adult life arising from trauma which, in a most healthy individuals, would not have resulted in a fracture? | 1 | 2 |
| Has either of your parents had a hip fracture? | 1 | 2 |

## Have you fallen during last three (3) months? (A fall is defined as an event which results in a person coming to rest inadvertently on the ground or floor or other lower level.)

1 No

2 Yes, _____ times

# senses

## Answer the following items based on the difficulties you experience in daily life using the assistive devices (eg. eyeglasses, hearing aid) that you usually use.

|  | Yes | No |
| --- | --- | --- |
| Do you have difficulties seeing far (eg. bus numbers) due to poor vision? | 1 | 2 |
| Do you have difficulties in close activities (eg. handcrafts, reading) due to poor vision? | 1 | 2 |
| Do you have difficulties hearing (eg. using the phone)? | 1 | 2 |

# Mood and life satisfaction

## Have you suffered psychological stress or acute disease in the past 3 months?

1 Yes

2 No

## Do you feel that your life at present is…?

1 very interesting

2 fairly interesting

3 fairly boring

4 very boring

5 I don't know

## Do you feel that your life at present is …?

1 very happy

2 fairly happy

3 fairly sad

4 very sad

5 I don't know

## Do you feel that your life at present is …?

1 very easy

2 fairly easy

3 fairly hard

4 very hard

5 I don't know

## Do you feel that at the present moment you are …?

1 very lonely

2 fairly lonely

3 not at all lonely

4 I don't know

## in the last month, how often have you …?

|  | Very often | Fairly often | Sometimes | Almost never | Never |
| --- | --- | --- | --- | --- | --- |
| … been upset because of something that happened unexpectedly? | 1 | 2 | 3 | 4 | 5 |
| … felt that you were unable to control the important things in your life? | 1 | 2 | 3 | 4 | 5 |
| … felt nervous and “stressed”? | 1 | 2 | 3 | 4 | 5 |
| … felt confident about your ability to handle your personal problems? | 1 | 2 | 3 | 4 | 5 |
| … felt that things were going your way? | 1 | 2 | 3 | 4 | 5 |
| … found that you could not cope with all the things that you had to do? | 1 | 2 | 3 | 4 | 5 |
| … been able to control irritations in your life? | 1 | 2 | 3 | 4 | 5 |
| … felt that you were on top of things? | 1 | 2 | 3 | 4 | 5 |
| … been angered because of things that were outside of your control? | 1 | 2 | 3 | 4 | 5 |
| … felt difficulties were piling up so high that you could not overcome them? | 1 | 2 | 3 | 4 | 5 |

# Personality and temperament

## Please answer the following questions about yourself by indicating the extent of your agreement. Please be as honest and accurate as you can throughout.  Try not to let your response to one statement influence your responses to other statements.  There are no "correct" or "incorrect" answers.  Answer according to your own feelings, rather than how you think "most people" would answer.

|  | Strongly agree | Agree | Neutral | Disagree | Strongly disagree |
| --- | --- | --- | --- | --- | --- |
| In uncertain times, I usually expect the best. | 1 | 2 | 3 | 4 | 5 |
| If something can go wrong for me, it will. | 1 | 2 | 3 | 4 | 5 |
| I'm always optimistic about my future. | 1 | 2 | 3 | 4 | 5 |
| I hardly ever expect things to go my way. | 1 | 2 | 3 | 4 | 5 |
| I rarely count on good things happening to me. | 1 | 2 | 3 | 4 | 5 |
| Overall, I expect more good things to happen to me than bad thing | 1 | 2 | 3 | 4 | 5 |
| I am easily irritated. | 1 | 2 | 3 | 4 | 5 |
| I have trouble controlling my temper. | 1 | 2 | 3 | 4 | 5 |

## The following questions are about the way you feel and act. instead of thinking too long, Please answer each question by SELECTING with circle (❍) THE ANWER that best describes your first feelings (please only select one ANSWER per row)

|  | Yes | No |
| --- | --- | --- |
| Do you like to have lots of things going on around you?? | 1 | 2 |
| Do you almost always have an answer ready when spoken to? | 1 | 2 |
| Do you prefer to keep to the background in the company of people? | 1 | 2 |
| Do you regard yourself as happy and carefree? | 1 | 2 |
| Do you have a lively manner? | 1 | 2 |
| Can you quickly describe your thoughts in words? | 1 | 2 |
| Do you have anything against selling things or asking people for money for some charitable purpose? | 1 | 2 |
| Do you keep things to yourself except with good friends? | 1 | 2 |
| Do you like to crack jokes and tell funny stories to your friends? | 1 | 2 |

# health information

## what is your opinion on the following statements?

|  | Yes | No | Not sure |
| --- | --- | --- | --- |
| It is difficult to influence your health on your own | 1 | 2 | 3 |
| I am often worried about my health | 1 | 2 | 3 |
| I feel that my health is to a high degree influenced by chance | 1 | 2 | 3 |

## what is your opinion on the following statements?

|  | Strongly agree | Moderately agree | Neither agree nor disagree | Moderately disagree | Strongly disagree |
| --- | --- | --- | --- | --- | --- |
| I like to get health information from a variety of sources | 1 | 2 | 3 | 4 | 5 |
| It is easy to assess the reliability of health information on the Internet | 1 | 2 | 3 | 4 | 5 |
| Terms and sentences of health information are often difficult to understand | 1 | 2 | 3 | 4 | 5 |
| It is difficult to know who to believe in health issues. | 1 | 2 | 3 | 4 | 5 |

## Next statements are about health related information e.g., in magazines or in Internet:

|  | Yes | No |
| --- | --- | --- |
| Health related information is presented interestingly | 1 | 2 |
| Health related stories and articles are too long | 1 | 2 |
| Health related stories and articles are often too scientific | 1 | 2 |
| Experts often disagree on what is healthy and what is not | 1 | 2 |
| Health related information in magazines and in Internet is mainly addressed to young persons | 1 | 2 |
| Information about the improvement of my physical fitness motivates me | 1 | 2 |
| The information given by different kinds of monitors, e.g., pedometer or heart rate monitor, motivates me | 1 | 2 |
| I get motivated when I have done better than others | 1 | 2 |
| I desire scientific facts about health information | 1 | 2 |

## On which health topics do you need health information? Please select 1-3 most important for you.

| Exercise | ☐ |
| --- | --- |
| Nutrition and diet | ☐ |
| Prevention of diseases | ☐ |
| Symptoms of a specific disease | ☐ |
| Treatment of a specific disease | ☐ |
| Medication | ☐ |
| Injury related issues | ☐ |
| Something else, what | ☐ |
| I don't need health information | ☐ |

## In the last month, have you...

|  | Yes | No |
| --- | --- | --- |
| …searched for information related to exercise? | 1 | 2 |
| …discussed with others about issues related to exercise? | 1 | 2 |
| …told to others what you have heard or read about exercise? | 1 | 2 |
| …avoided thinking about exercise? | 1 | 2 |
| …avoided information related to exercise? | 1 | 2 |

# Barriers and enablers of physical activity

## Do the following items limit your physical activities in your free time?

|  | Yes | No |
| --- | --- | --- |
| Lack of interest | 1 | 2 |
| Lack of time | 1 | 2 |
| Laziness | 1 | 2 |
| Exhaustion due to work, studying, etc. | 1 | 2 |
| Depressed mood or general exhaustion | 1 | 2 |
| Lack of skills | 1 | 2 |
| I don't know how to exercise | 1 | 2 |
| Illness or injury | 1 | 2 |
| Lack of money | 1 | 2 |
| Lack of exercise location | 1 | 2 |
| Lack of exercise equipment | 1 | 2 |
| Bad commuting to exercise locations | 1 | 2 |
| Lack of a suitable form of physical activity | 1 | 2 |
| Lack of directed physical activity | 1 | 2 |
| Lack of a suitable group | 1 | 2 |
| Exercise makes me feel uncomfortable | 1 | 2 |
| Shame | 1 | 2 |
| I can't find enough information on hobbies related to nature | 1 | 2 |
| Seasons (winter) | 1 | 2 |
| Other, what? | 1 | 2 |

# Nutrition and weight

## To your knowledge, were/are you at

|  | Underweight | Normal weight | Overweight |
| --- | --- | --- | --- |
| Age 15 | 1 | 2 | 3 |
| Age 30 | 1 | 2 | 3 |
| Age 50 | 1 | 2 | 3 |
| Currently | 1 | 2 | 3 |

## Over the past three months, have you eaten less than normally due to lack of appetite, chewing, or swallowing difficulties?

1 Severe decrease in food intake

2 Moderate decrease in food intake

3 No decrease in food intake

## which of the following statements describe your relationship to eating?

|  | Yes | No |
| --- | --- | --- |
| Do you make yourself sick (vomit) because you feel uncomfortably full?? | 1 | 2 |
| Do you worry you have lost control over how much you eat? | 1 | 2 |
| Do you believe yourself to be fat when others say you are thin? | 1 | 2 |
| Would you say food dominates your life? | 1 | 2 |

# Unsafety

## Do you feel unsafe?

|  | Yes | No |
| --- | --- | --- |
| At home? | 1 | 2 |
| Outside home? | 1 | 2 |

## Have you experienced physical, psychical or sexual violence or abuse (please select all that apply)

|  | Yes | No |
| --- | --- | --- |
| When you were under 18 | 1 | 2 |
| In your adult life | 1 | 2 |
| Within the last year | 1 | 2 |

# gaming

## In the last 12 months, have you played the following kinds of games either traditionally or on a mobile phone, computer or game console? (select all that apply)

|  | Traditionally | On a computer, phone or other other home device | I haven't played |
| --- | --- | --- | --- |
| Quizzes? (e.g. Trivial Pursuit) | 1 | 2 | 3 |
| Crosswords or sudokus? | 1 | 2 | 3 |
| Card games? (e.g. solitaire) | 1 | 2 | 3 |
| Gambling (lottery, slots, bingo)? | 1 | 2 | 3 |
| Board games? | 1 | 2 | 3 |
| Billiards/corona? | 1 | 2 | 3 |
| Exercise games (eg. petanque, Wii Fit)? | 1 | 2 | 3 |
| Strategy games (eg. chess, computer strategy games)? | 1 | 2 | 3 |
| Other games, what? | 1 | 2 | 3 |

## If you have played games, do you play

|  | Yes | No |
| --- | --- | --- |
| Alone | 1 | 2 |
| With friends | 1 | 2 |

## If you have played games, why did you play?

|  | Yes | No | Not sure |
| --- | --- | --- | --- |
| Gaming is relaxing | 1 | 2 | 3 |
| Gaming is fun | 1 | 2 | 3 |
| Gaming is a good way to spend time | 1 | 2 | 3 |
| Games are challenging | 1 | 2 | 3 |
| Games train (mental/physical) skills | 1 | 2 | 3 |
| Other reason, what: | | | |

## If you haven't played games, why haven't you?

|  | Yes | No | Not sure |
| --- | --- | --- | --- |
| Games aren't interesting | 1 | 2 | 3 |
| Games aren't meant for people my age | 1 | 2 | 3 |
| Insufficient skills | 1 | 2 | 3 |
| There are no games that suit me | 1 | 2 | 3 |
| There is no benefit in playing games | 1 | 2 | 3 |
| Other reason, what: | | | |

## Which of the following properties most intrigue you in games? Number them in order of importance 1-4, where 1 is the most important.

|  | Importance 1-4 (1 is most important, 4 is least important) |
| --- | --- |
| Accumulating scores and other achievements? |  |
| Discovering and exploring new things and features? |  |
| Fighting, combat and excitement, winning over other players / computer adversaries? |  |
| Interaction with other players / the story of the game / characters in the game? |  |

## What games do you play on the mobile phone, computer or game console?

_____________________________________________________________________________________________________________________________________________________________________________________________________________________________________________________________________________________________________________________________________

# Use of information technology

## Do you or anyone in your household have access to the Internet at home? (by any device)

1 Yes

2 No

3 I don’t know

## How often have you used internet during the last 3 months in average?

1 Daily or almost daily

2 At least once per week

3 Less than once a week

4 Not at all

## For what purposes have you used the Internet within the last year (12 months)?

|  | Yes | Rarely | No, but I would be interested | No |
| --- | --- | --- | --- | --- |
| eServices (eg. online banking, taxes, tickets, social services) | 1 | 2 | 3 | 4 |
| Finding infomation (eg. schedules, health information, yellow pages, recipes) | 1 | 2 | 3 | 4 |
| Following the news | 1 | 2 | 3 | 4 |
| Keeping in touch with friends (eg. email, Skype) | 1 | 2 | 3 | 4 |
| Making new friends | 1 | 2 | 3 | 4 |
| Using entertainment such as movies, books, or music | 1 | 2 | 3 | 4 |
| Shopping | 1 | 2 | 3 | 4 |
| I have my own home page or blog | 1 | 2 | 3 | 4 |
| Social media (eg. Facebook, Twitter) | 1 | 2 | 3 | 4 |
| I follow discussion forums | 1 | 2 | 3 | 4 |
| I actively post on discussion forums | 1 | 2 | 3 | 4 |
| I produce other online content, what? | 1 | 2 | 3 | 4 |
| Hobbies or studies | 1 | 2 | 3 | 4 |
| Gaming | 1 | 2 | 3 | 4 |
| Work | 1 | 2 | 3 | 4 |
| Other, what? | | | | |

## In the last 12 months have you used following devices:

|  | Yes, no major difficulties | Yes, difficult to do | No | I don’t know |
| --- | --- | --- | --- | --- |
| Computer | 1 | 2 | 3 | 4 |
| A simplified computer for the elderly | 1 | 2 | 3 | 4 |
| Tablet computer | 1 | 2 | 3 | 4 |
| Mobile phone | 1 | 2 | 3 | 4 |
| A simplified mobile phone for the elderly | 1 | 2 | 3 | 4 |
| Mobile phone with touch screen | 1 | 2 | 3 | 4 |

## Have you ever done one of the FOLLOWING?

|  | Yes, no major difficulties | Yes, difficult to do | No | I don’t know |
| --- | --- | --- | --- | --- |
| Installed a new program on a phone or tablet? | 1 | 2 | 3 | 4 |
| Determined your location with a phone or other device (GPS)? | 1 | 2 | 3 | 4 |
| Made or received a video call (videophone or messaging eg. Skype)? | 1 | 2 | 3 | 4 |
| Used the Internet on a mobile phone? | 1 | 2 | 3 | 4 |
|  |  |  |  |  |

## Tell your opinions on the following statements, which are related to the use of portable information technologies (IT) such as smartphone of tablet

|  | Yes | No | I don’t know |
| --- | --- | --- | --- |
| I am interested in using portable information technologies (IT). | 1 | 2 | 3 |
| I believe using IT is/would be useful in my daily life. | 1 | 2 | 3 |
| I believe using IT is/would be necessary in my daily life. | 1 | 2 | 3 |
| Using IT is easy for me. | 1 | 2 | 3 |
| Using IT is too expensive for me. | 1 | 2 | 3 |
| I have privacy or security concerns regarding the use of IT. | 1 | 2 | 3 |
| Other people think that I should use IT. | 1 | 2 | 3 |
| Other reasons why I use or don't use IT: | | | |

# Use of exercise technology

## Which of the following have you used to track your physical activity?

|  | Yes | No | I don’t know |
| --- | --- | --- | --- |
| Pedometer (step counter) | 1 | 2 | 3 |
| Heart rate monitor | 1 | 2 | 3 |
| Mobile phone | 1 | 2 | 3 |
| Exercise diary (paper) | 1 | 2 | 3 |
| Exercise diary (electric) | 1 | 2 | 3 |
| Wrist-worn activity monitor | 1 | 2 | 3 |
| Waist-worn activity monitor | 1 | 2 | 3 |
| Other, what: | 1 | 2 | 3 |

## Tell your opinions on the following statements, which are related to the use of electronic activity monitors such as step counter or heart rate monitor

|  | Yes | No | I don’t know |
| --- | --- | --- | --- |
| I am interested in using activity monitors. | 1 | 2 | 3 |
| I believe using activity monitors is/would be useful for me. | 1 | 2 | 3 |
| I believe using activity monitors is/would be necessary in my daily life. | 1 | 2 | 3 |
| Using activity monitors is easy for me. | 1 | 2 | 3 |
| Activity monitors are too expensive for me. | 1 | 2 | 3 |
| Other people think that I should use activity monitors. | 1 | 2 | 3 |
| Other reasons why I use or don't use activity monitors: | | | |

# Sleep

|  | Good | Satisfractory | Poor |
| --- | --- | --- | --- |
| How do you feel the amount of sleep you currently have? | 1 | 2 | 3 |
| How do you feel the quality of sleep you currently have (regardless of how much you sleep)? | 1 | 2 | 3 |

# Nature relationship, exercise environment, nature environment in town area

## The following questions are about your relationship with nature. Please answer each question by crossing the answer circle (❍) that best describes your feelings (please only select one circle per row)

|  | Completely agree | Somewhat agree | Neither agree nor disagree | Somewhat disagree | Completely disagree |
| --- | --- | --- | --- | --- | --- |
| I enjoy being outdoors, even in bad weather. | 1 | 2 | 3 | 4 | 5 |
| My ideal nature resort is in the middle of nature, far from civilization. | 1 | 2 | 3 | 4 | 5 |
| I always think of how my actions influence the environment. | 1 | 2 | 3 | 4 | 5 |
| Nature inspires me and gives one purpose to my life. | 1 | 2 | 3 | 4 | 5 |
| I have been active in nature ever since I was a child. | 1 | 2 | 3 | 4 | 5 |
| Thinking of being deep in the woods, far from civilization, is frightening. | 1 | 2 | 3 | 4 | 5 |
| I notice ambient nature even if I'm in the middle of the city. | 1 | 2 | 3 | 4 | 5 |
| My relationship to nature is an important part of who I am. | 1 | 2 | 3 | 4 | 5 |
| Nature is an important part of my wellbeing. | 1 | 2 | 3 | 4 | 5 |

# Fear of falling

## How concerned are you about the possibility of falling while doing the below activities? If you currently don’t do the activity, please answer to show whether you think you would be concerned about falling IF you did the activity.

|  | Not at all concerned | Somewhat concerned | Fairly concerned | Very concerned |
| --- | --- | --- | --- | --- |
| Getting dressed or undressed | 1 | 2 | 3 | 4 |
| Taking a bath or shower | 1 | 2 | 3 | 4 |
| Getting in or out of a chair | 1 | 2 | 3 | 4 |
| Going up or down stairs | 1 | 2 | 3 | 4 |
| Reaching for something above your head or on the ground | 1 | 2 | 3 | 4 |
| Walking up or down a slope | 1 | 2 | 3 | 4 |
| Going out to a social event (e.g. religious service, family gathering or club meeting) | 1 | 2 | 3 | 4 |

# Social environment

## Please indicate for each of the statements, the extent to which they apply to your situation, the way you feel now?

|  | Definitely yes | Yes | More or less | No | Definitely no |
| --- | --- | --- | --- | --- | --- |
| I experience a general sense of emptiness. | 1 | 2 | 3 | 4 | 5 |
| There are plenty of people I can rely on when I have problems. | 1 | 2 | 3 | 4 | 5 |
| There are many people I can trust completely. | 1 | 2 | 3 | 4 | 5 |
| There are enough people I feel close. | 1 | 2 | 3 | 4 | 5 |
| I miss having people around. | 1 | 2 | 3 | 4 | 5 |
| I often feel rejected. | 1 | 2 | 3 | 4 | 5 |

## Do you participate in the activities of a club, union, society, hobby group or spiritual or religious society (eg. sports group, political party, choir, congregation)?

1 No
2 Yes, actively
3 Yes, occasionally

# Other

## i filled this form

1 myself (or with my companion)
2 with the help of other next of kin, or practical nurse
